# Supplementary material for: Degradability and Properties of PBAT-Based Biodegradable Mulch Films in Field and Their Effects on Cotton Planting
Source: Polymers (Basel). 2022 Aug 2;14(15):3157. doi: 10.3390/polym14153157 (PMC9371060; doi:10.3390/polym14153157)
Supplement: Supplementary file 1 [file polymers-14-03157-s001.zip › polymers-1804790-supplementary.pdf]

Supplementary Materials: Degradability and Properties of PBAT-Based Biodegradable Mulch Films in Field and Their Effects on Cotton Planting

Qi Liu [1], Yue Wang [1], Jialei Liu [1], Xiaowei Liu [2], Yanyan Dong [1], Xin Huang [1], Zhichao Zhen [3,*], Jun Lv [2,*] and Wenqing He [1,*]

[1] National Engineering Laboratory for Crop Efficient Water Use and Disaster Mitigation, Key Laboratory of Prevention and Control of Residual Pollution in Agricultural Film, Ministry of Agriculture and Rural
Affairs, Institute of Environment and Sustainable Development in Agriculture, Chinese Academy of
Agricultural Sciences, Beijing, 100081, China; liuqi@caas.cn (Q.L.); wyw111y@sina.com (Y.W.);
liujialei@caas.cn (J.L.); dongyanyan@caas.cn (Y.D.); huangxinharris@sina.com (X.H.)

[2] Shihezi Agricultural Science Research Institute, Xinjiang 832000, China; liuxiaowei1212@sina.com

[3] Technical Institute of Physics and Chemistry, Chinese Academy of Sciences, Beijing 100045, China

* Correspondence: zhenzc1979@163.com (Z.Z.); lvjunshznky@sina.com (J.L.); hewenqing@caas.cn (W.H.)

Table S1. Basic agricultural application performances of the films for mulching.

Samples	Base material	Thickness (μm)	Light Transmittance (%)	Dislocation rate of Mechanical Perforating (%)
BDM	PBAT[a]	10.2 ± 0.1	90.48 ± 1.34	91.79
BDM1	PBAT[a]	10.3 ± 0.3	88.08 ± 2.41	91.93
BDM2	PBAT[a]	9.9 ± 0.1	76.95 ± 4.72	91.00
PE	Polyethylene	8.9 ± 0.6	93.20 ± 0.87	91.53

[a] AbbreviationsofPoly(butylene adipate-co-terephthalate).

Table S2. Basic physical and chemical properties of soil in the field-test site.

Items	Soil bulk density ($g\cdot cm^{-3}$)	Organic matter ($g\cdot kg^{-1}$)	Available nitrogen ($mg\cdot kg^{-1}$)	Rapidly available phosphorus ($mg\cdot kg^{-1}$)	Rapidly available potassium ($mg\cdot kg^{-1}$)	pH
**Results**	1.38 ± 0.05	30.41 ± 7.71	69.33 ± 24.32	26.22 ± 8.68	299.94 ± 36.11	7.87 ± 0.14

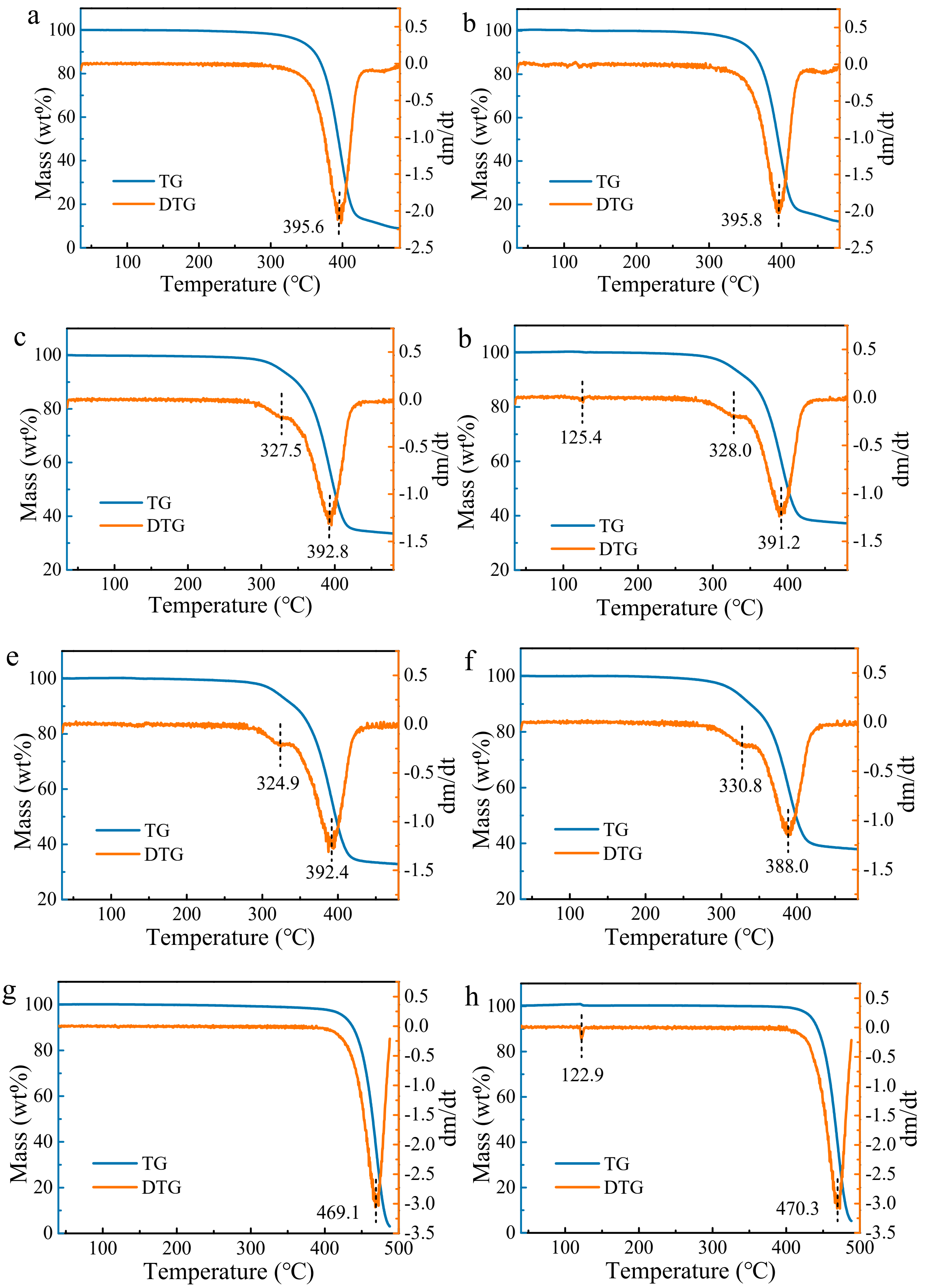
a
Mass (wt%)
dm/dt
Temperature (°C)
TG
DTG
395.6
b
Mass (wt%)
dm/dt
Temperature (°C)
TG
DTG
395.8
c
Mass (wt%)
dm/dt
Temperature (°C)
TG
DTG
327.5
392.8
b
Mass (wt%)
dm/dt
Temperature (°C)
TG
DTG
125.4
328.0
391.2
e
Mass (wt%)
dm/dt
Temperature (°C)
TG
DTG
324.9
392.4
f
Mass (wt%)
dm/dt
Temperature (°C)
TG
DTG
330.8
388.0
g
Mass (wt%)
dm/dt
Temperature (°C)
TG
DTG
469.1
h
Mass (wt%)
dm/dt
Temperature (°C)
TG
DTG
122.9
470.3

Figure S1. TG and DTG curves of BDM (a), BDM’ (b), BDM1 (c), BDM1’ (d), BDM2 (e), BDM2’(f), PE (g), and PE’(h).

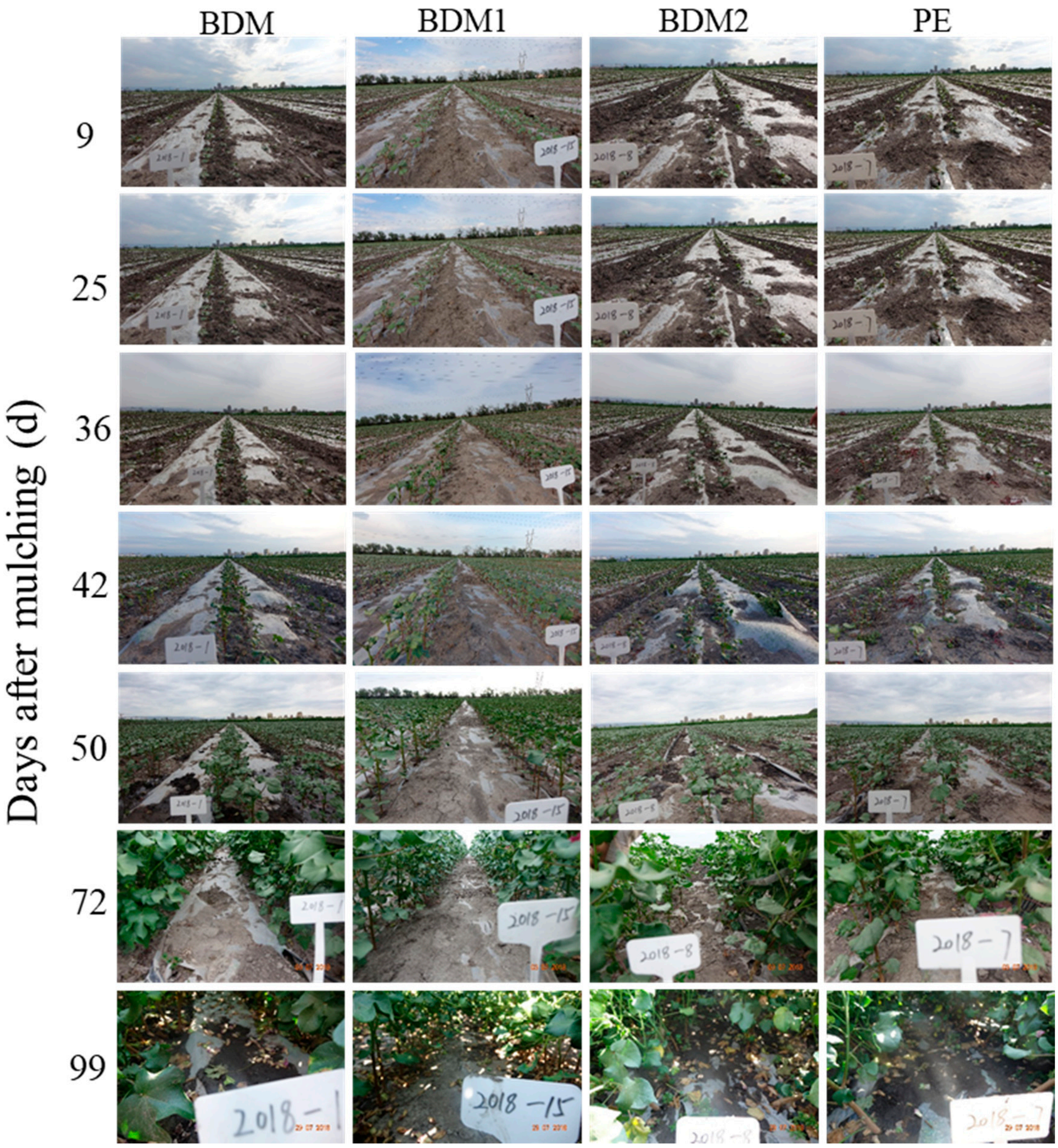

Figure S2. Degradation digital photos along a line of mulched cotton of BDM, BDM1, BDM2 and PE films during mulching period.
